# Aging dysregulates D- and E-series resolvins to modulate cardiosplenic and cardiorenal network following myocardial infarction

**DOI:** 10.18632/aging.101077

**Published:** 2016-10-18

**Authors:** Ganesh V. Halade, Vasundhara Kain, Laurence M. Black, Sumanth D. Prabhu, Kevin A. Ingle

**Affiliations:** ^1^ Division of Cardiovascular Disease, Department of Medicine, The University of Alabama at Birmingham, AL 35233, USA

**Keywords:** aging, non-resolving inflammation, lipid mediators, lipoxygenase, macrophages, myocardial infarction

## Abstract

Post-myocardial infarction (MI), overactive inflammation is the hallmark of aging, however, the mechanism is unclear. We hypothesized that excess influx of omega 6 fatty acids may impair resolution, thus impacting the cardiosplenic and cardiorenal network post-MI. Young and aging mice were fed on standard lab chow (LC) and excess fatty acid (safflower oil; SO)-enriched diet for 2 months and were then subjected to MI surgery. Despite similar infarct areas and left ventricle (LV) dysfunction post-MI, splenic mass spectrometry data revealed higher levels of arachidonic acid (AA) derived pro-inflammatory metabolites in young-SO, but minimal formation of docosanoids, D- and E- series resolvins in SO-fed aged mice. The aged mice receiving excess intake of fatty acids exhibit; 1) decreased lipoxygenases (5-,12-, and 15) in the infarcted LV; 2) lower levels of 14HDHA, RvD1, RvD5, protectin D1, 7(S)maresin1, 8-,11-,18-HEPE and RvE3 with high levels of tetranor-12-HETEs; 3) dual population of macrophages (CD11b^low^/F480^high^ and CD11b^high^/F480^high^) with increased pro-inflammatory (CD11b^+^F4/80^+^Ly6C^hi^) phenotype and; 4) increased kidney injury marker NGAL with increased expression of TNF-ɑ and IL-1β indicating MI-induced non-resolving response compared with LC-group. Thus, excess fatty acid intake magnifies the post-MI chemokine signaling and inflames the cardiosplenic and cardiorenal network towards a non-resolving microenvironment in aging.

## INTRODUCTION

Heart failure (HF) is an enormous medical burden and the leading cause of mortality in aging populations [1]. Nearly 2% of the US population, almost 5 million people, are affected with HF as an age-related disease following myocardial infarction (MI) [1, 2]. Post-MI, the acute response is from the spleen to direct early healing, but the chronic supply of neutrophils and monocyte/macrophages triggers the non-resolving response that progresses to HF [3-6]. Perturbed or dysregulated healing post-MI is characterized by marked inflammation and coronary syndrome pathology, leading to non-resolving inflammation [5, 7-9]. Lipoxygenase (LOX) enzymes remodel fatty acids and program the non-resolving or resolving inflammation response post-MI, thus the enzyme-substrate availability or substrate imbalance defines the formation of pro-inflammatory or resolving mediator(s) post-MI [5, 8]. With application of mass spectrometry-based structural elucidation, it is now validated that omega-3 and omega-6 fatty acids are biosynthetic precursors of resolvins, maresin, protectins and lipoxins, with inflammation-resolving properties [10]. Omega-6 plays an essential role in health homeostasis, however, we found that chronic, excessive intake of omega-6 leads to progression of non-resolving inflammation in obese aging mice, post-MI and development of bone marrow adiposity [5, 11]. Recent evidence suggests that MI-induced events are a major sign of metabolic disarray in the LV healing with limited generation of resolving lipid mediators, resulting in non-resolving inflammation, though the mechanism(s) is unclear. [5, 12, 13]. Several studies have explored derangements of glucose and fatty acids to define the role of lipotoxicity, glucotoxicity, or glucolipotoxicity in HF progression [14]. Proteomics and metabolomics databases indicate that substrate variability, such as fatty acids, amino acids, protein, and glucose, regulates cardiac remodeling following MI [12]. The fundamental limitation of the currently published knowledge is the lack of data in regards to which specific pathway to target for enzyme(s) individually or grouped. This information could be essential for discovering therapeutic treatments for the millions of aging patients at risk of HF post-MI to resolve their inflammation and potentially halt the life threatening progression to HF.

LOXs (-5, -12 and -15) are lipid-busting enzymes that use fatty acids to synthesize bioactive molecules in response to myocardial stress or injury [8, 15]. Post-MI, LOX-derived specialized mediators/biomolecules facilitate immune cell phenotypes, residual time at the site of injury and their kinetics in order to repress or progress inflammation to modulate LV healing [5, 8]. Data has suggested that LOXs are increased in the failing heart [13], but the details of how excess intake of omega-6 fatty acids affects resolving/non-resolving bioactive(s) are unclear. Excess intake of omega-6 not only alters bone marrow myeloid milieu, but also decreases lifespan, aggravates non-resolving inflam-mation, and develops nephropathy in lupus-prone inflamed mice, [11, 16] therefore the present report investigated the cardiorenal network in HF pathology. To investigate the chemical nature of cardiorenal HF and to integrate the translational outlook of obesity superimposed on aging, we determined the fatty acids involved in remodeling post-MI in young adults (6 month) and aging (18 month) mice using liquid chromatography–mass spectrometry (LC-MS/MS) metabololipidomics methodology. Here, we hypo-thesized that excess intake of omega-6 alters the balance of pro-inflammatory and pro-resolving metabolites post-MI in aging, thereby driving non-resolving inflammation in the cardiosplenic and cardiorenal networks. Post-MI, in highly oxidative settings, an excess influx of omega-6 fatty acids reduces primarily D- (RvD1, RvD5) and E- (RvE3) series resolvins, 7(S) maresin, protectin D1, and other resolving precursors, such as 4-, 10-, 14-HDHA and 8-,11-,18- HEPE during the acute inflammatory phase and are essential to the pro-resolving response. Thus, an imbalance of these mediators resulted in the accumulation of pro-inflammatory macrophages (CD11b^+^F4/80^+^Ly6C^hi^) in the infarcted LV, leading to signs of MI-induced non-resolving inflammation. Despite the positive benefit of omega-6 fatty acids in cardiovascular health, excess or imbalanced intake dysregulates LOXs in the infarcted LV, leading to an insufficiency of resolving bioactives and, thereby, impaired resolution. Thus, post-MI, the non-resolving or pro-inflammatory macrophage phenotype depends on the eicosanoids and doco-sanoids present in the transcellular splenic milieu, interlinked with lipid signaling, inflammation, and LOXs activity.

## RESULTS

### MI-induced LV dysfunction and excess fatty acids reduced LOXs post-MI superimposed on aging

**Figure 1:**
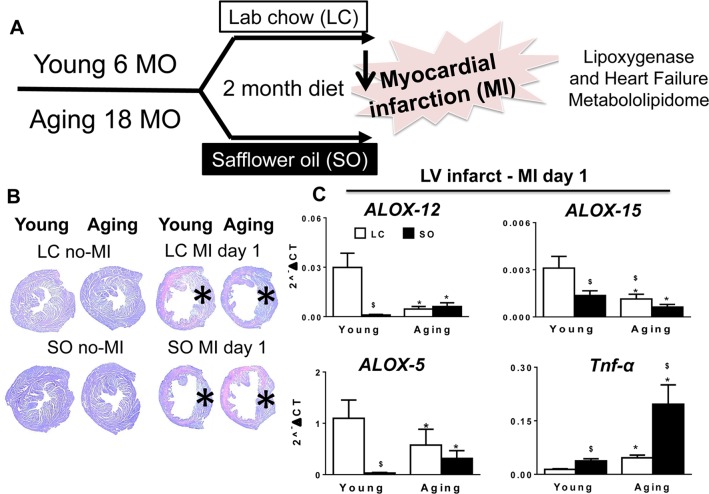
Excess fatty acid influx depleted LOXs in young and aging post-MI (A) Study design indicating young (6 months) and aging (18 months) mice on an omega-6 fatty acids enriched safflower oil diet protocol. (B) No-MI naïve control and infarcted LV stained with periodic acid-Schiff (PAS) at d1 post-MI in young and aging, with and without fatty acid enriched diet. No-MI control represents the steady state naïve control mice (C) mRNA expression of *LOXs (5,12,15)* and *TNF-ɑ* in infarcted LV. *p<0.05 vs young-LC; $ p<0.05 LC vs SO. Values are means ±SEM; n=2-4/group.

The robust post-MI LV chamber dilation response and wall thinning was observed within 24 hr in young and aging mice (Figure 1). These changes were marked by reduced LV fractional shortening at day (d) 1 in young and aging mice compared to naïve controls (Table 1). Post-MI, infarcted LV wall thinning was characterized by loss of glycogen content (pink area) using periodic acid schiff staining (PAS) compared to d0 naïve controls (Figure 1B). MI-induced compensatory hypertrophy of remote myocardium thickening was notable in young mice, but relatively less in SO-aging with marked thinning. The infarcted zone of necrotic myocardium was resorbed in all four groups post-MI. At the gene expression level, the aging mice on normal diet reduced level of LOXs (5-, 12-, and 15-) mRNA expression compared with young mice on standard diet. Further, excess intake of fatty acids in both young and aging mice displayed a decrease in LOX-15 and LOX-5 compared with respective controls on standard LC diet. Excess fatty acid diet in young mice reduced LOX-12 expression compared with their aged match control on normal diet, however no change was observed in LOX-12 expression in aging mice fed either normal or high fatty acid diet (Figure 1C). Since leukocyte derived pro-inflammatory cytokines contribute to the development of the inflammatory response post-MI, we measured TNF-α levels. The TNF-α levels were increased as an impact of aging and due to excess omega-6 fatty acids intake. Aging mice on SO diet displayed (4.5 fold; p<0.5) increase in TNF-α expression compared with young-SO cohorts (Figure 1C). Collectively, excess influx of fatty acids in aging reduced LOX expression with increased inflammation in LV healing post-MI.

**Table 1:** Necropsy and echocardiography parameters indicating reduced LV remodeling, pulmonary edema and LV function in young and aged SO-fed and LC-fed mice post-MI

	No-MI				MI day 1			
Parameters	Young-LC (n=19)	Young-SO (n=13)	Aging-LC (n=11)	Aging-SO (n=13)	Young-LC (n=7)	Young-SO (n=9)	Aging-LC (n=9)	Aging-SO (n=14)
Necropsy parameters								
Body weight (g)	34 ± 1	31 ± 1	36 ± 2	34 ± 1	29 ± 1	30 ± 1	35 ± 3	35 ± 1
LV (mg)	74± 5	62 ± 2	114 ± 6	99 ± 3	108 ± 5	88 ± 3	115 ± 6	105 ± 4
LV/BW:LV/Tibia (mg/g)	0.6 ± 0.01	0.6 ± 0.01	0.7 ± 0.01	0.8 ± 0.03*	0.6 ± 0.01	0.6 ± 0.01*	0.5 ± 0.01	0.5 ± 0.1
RV/BW (mg/g)	0.6 ± 0.01	0.6 ± 0.01	0.6 ± 0.02	0.6 ± 0.01	0.7 ± 0.01	0.8 ± 0.02	0.6 ± 0.02	0.6 ± 0.01
Lung weight/Body weight (mg/g)	5.5 ± 1	5.4 ± 0.1	4.1 ± 0.2	4.7 ± 0.2	5.4 ± 0.1	5.8 ± 0.8	6.2 ± 1	6 ± 1
Tibia (mm)	17 ± 0.2	17 ± 0.2	17 ± 0.6	17 ± 0.4	17 ± 0.2	17 ± 0.1	17 ± 0.2	17 ± 0.2
Infarct area (%)	ND	ND	ND	ND	53 ± 2	53 ± 1	50 ± 1	49 ± 1
Echocardiography parameters								
Heart Rate (bpm)	452 ± 17	456 ± 20	473 ± 17	427 ± 7	450 ± 20	471 ± 20	461 ± 8	520 ± 12
EDV (ul)	82 ± 7	65 ± 5	72 ± 5	69 ± 4	79 ± 50	74 ± 4	89 ± 5	82 ± 5
ESV (ul)	35 ± 5	26 ± 2	35 ± 4	30 ± 3	65 ± 2*	58 ± 4*	74 ± 3*	67 ± 5*
Fractional Shortening %	30 ± 2	31 ± 1	27 ± 1	30 ± 2	8 ± 1*	10 ± 1*	7 ± 2*	11± 1*
PWTs (mm) Infarcted wall	0.67 ± 0.03	0.66 ± 0.04	0.71 ± 0.05	0.68 ± 0.04	0.48 ± 0.04*	0.50± 0.038	0.55 ± 0.068	0.57 ± 0.048

Values are means ± SEM. * p<0.05 vs young-LC, ‘n’ indicates sample size; LV, Left Ventricle; BW. Body weight; RV, Right Ventricle; - = No infarct in d0 naïve controls; bpm, beats per minute; EDV, end-diastolic volume; ESV, end-systolic volume; PWT, posterior wall thickness, S, systole; mm, millimeter

### Aging impacts cardiosplenic metabolome post-MI in excess fatty acids fed mice

**Figure 2:**
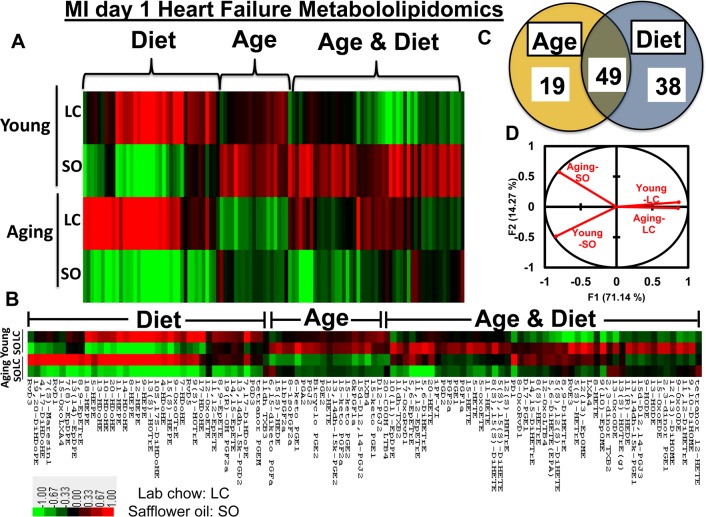
Aging and intake of fatty acids impacts metabololipidomics profiling in LV healing (**A** and **B**) Hierarchal cluster analyses of lipids indicates increased levels of metabolites in young but decreased in SO diet fed aging group post-MI. Color code bar representing change in expression from green (-1 lowest decrease) to red (+1 highest increase). (**C**) Venn diagram representing the number of metabolites affected due to age (young vs aging) and SO diet post-MI. (**D**) Principal component analysis (PCA) of lipid metabolites suggesting limited intake of fatty acids in young and aging (LC) mice respond similar manner post-MI; n =3/group.

Post-MI, fatty acids remodel into several bioactive molecules that temper the repression or progression of resolution of inflammation in HF pathophysiology [8, 10]. To understand how the fatty acids remodel into eicosanoids and docosanoids using LOXs in young and aging mice post-MI, we used an unbiased, targeted multiple reaction monitoring (MRM) LC-MS/MS approach to resolve the splenic metabolites. We analyzed 117 lipid analytes from young and aging mice, with and without fatty acid supplement diet, at d0 and post-MI d1. The main goal of the study was to focus on MI-induced inflammation and lipid remodeling (pathological). Therefore, we focused on post-MI lipid modifications, but to assess the bioavailability of diet enrichment and its impact on LV-splenic network, we analyzed limited pre-MI samples (physiological; Supplementary Table 1). As an impact of aging, 18% of eicosanoids were decreased post-MI. The excess fatty acid supplements led to a robust increase in the levels of eicosanoids both in young and aging cohorts before MI, while the eicosanoids were decreased globally post-MI (Figure 2A; heat map, Supplementary Table 1). Further, supplementation of excess fatty acids in aging mice led to decrease in 90% of eicosanoids level compared with young–SO mice. Post-MI d1, heat map cluster analysis and the Venn diagram (Figures 2A and B) showed that the nearly 106 eicosanoids are impacted by age and excess intake of fatty acids. A total of 19 eicosanoids were changed as a result of aging, however, excess fatty acid intake, irrespective of age, influenced 38 meta-bolites. The levels of 49 eicosanoids were changed due to combined effect of both aging and excess of fatty acid intake. Principal component analysis (PCA) for global changes showed that young and aging cohorts on standard LC diet were closely associated. The diverse response to MI is indicated by the differential lipidomics profiling in young and aging cohorts due to excess influx of excess omega-6 fatty acids Figure 2C-D).

### Aging dysregulated AA-derived metabolites post-MI

Prior to MI (d0), there was no difference observed between groups in the levels of arachidonic (AA) acid metabolites, 9,10-EpOME, PGA_2_, PGE_2,_ TXB_2_ and epoxyeicosatrienoic acids (EETs). The excess fatty acid intake impacted AA acid metabolism leading to increased levels 9,10-EpOME, PGA_2_, PGE_2,_ TXB_2_ and EETs in both young and aging mice (Supplementary Table 1).

**Figure 3:**
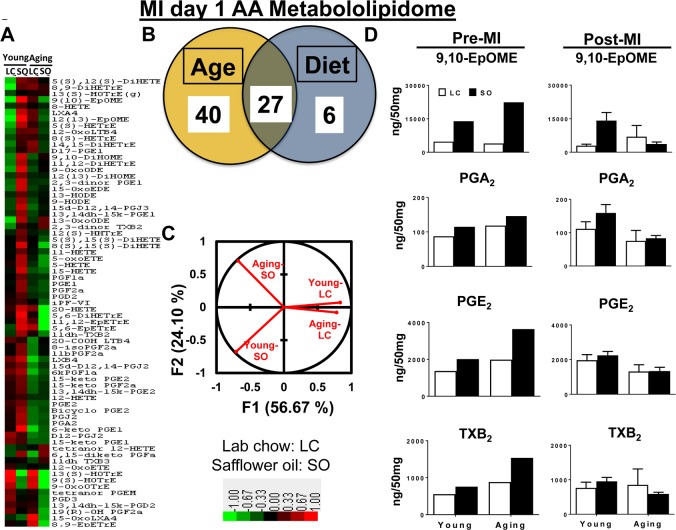
Excess fatty acids in aging decreases arachidonic acid (AA) metabololipidome post-MI (**A**) Hierarchal cluster analysis of change in AA metabolites due to young and aging with and without SO diet. Color code bar representing change in expression from green (-1 lowest decrease) to red (+1 highest increase). (**B**) Venn diagram representing the number of AA metabolites affected due to age (young and aging) and SO-diet post-MI. (**C**) PCA analysis of AA metabolites of post-MI with respect to age and diet. (**D**) Bar graph representing change in AA metabolite production at pre-MI (No-MI controls) and d1 post-MI.

Post-MI aging impacted the maximum number of eicosanoids that belongs to AA class. Overall levels of the respective eicosanoids decrease post-MI. Compared with young-LC mice, there was a further decrease in levels of eicosanoids in aging-LC mice. The excess intake of omega-6 fatty acid led to an increase in 9,10-EpOME and PGA_2_ in young-SO mice compared with young-LC mice, however was no change observed in levels of 9,10-EpOME, PGA_2_, PGE_2_ and TXB_2_ between aging-LC and -SO mice (Figure 3D). The heat map cluster analysis and Z score analyses (Supplementary Figure 5) showed that a total of 73 eicosanoids were changed post-MI as an impact of age and fatty acids. Nearly, ∼90% of eicosanoids decreased as a result of aging compared with young cohorts on normal diet. As an impact of excess intake of fatty acids, there was an increase in ~80% of eicosanoids in SO-fed young mice compared with young-LC post-MI. The aging mice did not display much difference between the levels of eicosanoids between LC and SO cohorts (Figure 3A). These changes are represented in the Venn diagram, which shows 40 AA eicosanoids were changed as a result of aging. Only 6 AA eicosanoids were influenced by the excess fatty acid intake and almost 27 AA eicosanoids were changed as result of both age and fatty acid (Figure 3B). Post-MI PCA analysis of young-LC, young-SO versus aging-LC, aging-SO MI groups showed a clear dissociation between excess fatty acid supplied young vs aging cohorts (Figure 3C): Z-score plots were constructed to identify metabolic changes distinct to each group in response to MI (Supplementary Figure 5). Thus, composition of lipid mediator milieu affects LV healing through reduced levels of LOXs expression and LOX-derived lipid mediators in post-MI setting.

### Excess intake of omega-6 fatty acids affects D-series resolvins, protectin and maresin pre and post-MI

**Figure 4:**
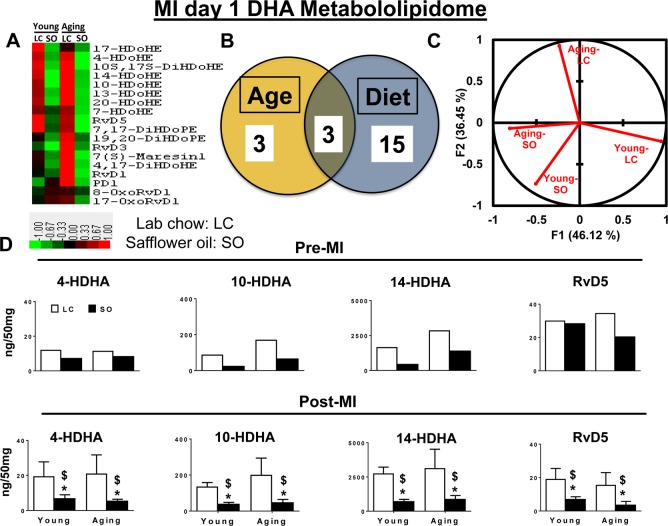
Excess omega-6 fatty acids dysregulate DHA metabololipidomics profile post-MI (**A**) Hierarchal cluster analysis of change in DHA metabolites in young and aging, with and without SO diet. Color code bar representing change in expression from green (-1 lowest decrease) to red (+1 highest increase). (**B**) Venn diagram representing the number of DHA metabolites affected due to age (young and aging) and SO diet post-MI. (**C**) PCA analysis of DHA metabolites with respect to age and diet post-MI. (**D**) Bar graph representing change in DHA metabolite production at pre-MI (No-MI controls) and d1 post-MI.

LOXs utilize omega-3 and omega-6 (arachidonic acid; 20:4n-6) as substrates in response to injury or infection to form bioactive molecules [5, 10]. Omega-3 and -6 fatty acids exert protective effects on cardiovascular system and their imbalance aggravates HF progression post-MI [17]. Therefore, we further analyzed the DHA metabolome in an excess of omega-6 environment. A total of 21 DHA metabolites were detected in the spleens of young and aging cohorts with excess omega-6 fatty acid intake. There were minimal difference was observed within the DHA metabolome when compared between young and aging cohorts on normal diet. As an impact of excess omega-6 intake, ~90% of DHA docosanoids decreased in both young and aging cohorts pre- and post-MI. The Venn diagram shows aging independently impacted 3 metabolites, while SO diet group showed changes in 15 eicosanoids, and age with excess fatty acid intake together impacted only 3 eicosanoids each (Figure 4A and B). Global PCA analyses of DHA docosanoids showed that both young-LC and aging-LC clearly dissociate from each other post-MI, but young-SO and aging-SO associate with each other (Figure 4C). Regulation of DHA docosanoids was diet-dependent in both pre- and post-MI condition setting. Young and aging SO supplement-ed group decreased D-series pro-resolving bioactives 4-,10-,14- HDHA, RvD5, 7(S)-maresin, protectin D1 (Figure 4D and Supplementary Table 1). Thus, excess intake of omega-6 fatty acids in aging affects the post-MI resolution of inflammation by decreasing D-series specialized pro-resolving bioactives.

### Excess intake of omega-6 fatty acids decreased E-series resolvins and their precursors

In relation to AA-derived eicosanoids and DHA derived-docosanoids, we also measured EPA-derived bioactive(s) in both young and aging cohorts pre- and post-MI. We found that none of the EPA eicosanoids were impacted due to aging in mice. A total of 7 eicosanoids were changed due to an excess influx of excess omega-6 intake. Age and diet both lead to the change in 5 eicosanoids (Supplementary Figure 3A and 3B, Supplementary Table 1). PCA analyses and biplot showed that all the four cohorts young-LC, young-SO, aging-LC and aging-SO are separated from each other (Supplementary Figure 3C and D, Supplementary Table 2). Similar to DHA docosanoids, ~90% of the EPA metabolome was decreased as a result of excess intake of omega-6 fatty acid in both young and aging cohorts post-MI. As shown in Supplementary Figure 3D, the pro-resolving 8-, 9-,11-, and 18-HEPE were significantly decreased in SO fed young and aging mice post-MI. The precursor of E-series resolvin and RvE3 were increased in young-SO group, but significantly decreased in aging-SO group. Thus, our results showed that excess intake of omega-6 fatty acids impacted the EPA metabolome in aging.

**Table 2:** Brief description of fat and kcal/g that were supplemented to young and aged mice for 2 months prior to MI and heart failure lipidome analysis

Groups	Standard Lab Chow (LC)	Safflower oil (SO)
Research diets	D10012M (AIN-93M)	D11102001
	g %	g %
Protein	14	15
Carbohydrate	73	66
Fat	4	10
Omega-3 fatty acids	0.011	0.015
Omega-6 fatty acids	3.1	6.63
Omega-9 fatty acids	0.9	1.34
Kcal/g	3.8	4.2

### Excess fatty acid enhanced LV Ly6C^high^ population in aging-SO post-MI

**Figure 5:**
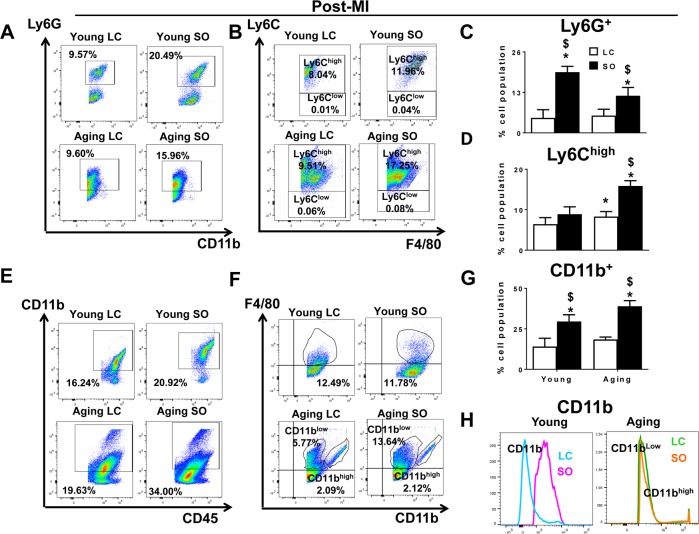
Excess intake of omega-6 fatty acids increased F4/80^+^/Ly6C^high^ Ly6G^+^, CD11b^+^ population post-MI (**A**) Representative dot plots identifying CD11b^+^ population in LV mononuclear cells isolated from LC and SO fed young mice at d1 post-MI. (**B**) Representative flow cytometry (FACs) dot plots showing Ly6C^high^ in LV mononuclear cells isolated from LC and SO fed young and aging mice at d1 post-MI. (**C**) Bar graphs representing percentage of Ly6G^+^ population in LV mononuclear cells at d1 post-MI. (**D**) Bar graphs representing percentage of Ly6C^high^ population in LV mononuclear cells at d1 post-MI. (**E**) Representative FACs dot plots showing CD45^+^/CD11b^+^ in LV mononuclear cells isolated from LC and SO fed young and aging mice at d1 post-MI. (**F**) Representative FACs dot plots showing CD11b^low^/F4/80^high^ and CD11b^high^/F4/80^high^ in LV mononuclear cells isolated from LC and SO fed young and aging mice at d1 post-MI. (**G**) Bar graphs representing percentage of CD11b^+^ population in LV mononuclear cells at d1 post-MI. (**H**) Histogram representing change in CD11b expression in young and aging mice post-MI. *p<0.05 vs young-LC; $ p<0.05 LC vs SO. n=3-5 mice/group for flow cytometry analysis.

Post-MI, neutrophil and macrophage infiltration into the infarcted LV dominates the early phase. There was no difference observed in the neutrophil population between the young and aging mice maintained on LC diet, but the SO enriched group (young and aging) displayed recruitment of excess neutrophils (CD11b^+^/Ly6G^+^). The SO-fed groups showed a robust increase the density of Ly6G^+^ cells in young (19.5±1.8%) and aging (11.9±2.6%) mice post-MI d1. Of note, the young-SO mice also displayed a higher expression of CD11b^+^/Ly6G^+^ cells compared with young-LC post-MI. No change was observed with expression levels of CD11b^+^/Ly6G^+^ within aging-LC and aging-SO mice post-MI (Figure 5A and 5C). In line with previous post-MI leukocyte kinetics, the monocytes differentiate to macrophages at the site of injury; aging-LC mice showed slightly higher percentage of CD11b^+^/F4/80^+^/Ly6C^high^ (8.3±1.2%) population compared to young-LC mice (6.4±1.6%) at post-MI d1 (Figure 5B). The Ly6C^high^ population at d1 post-MI was robustly increased in excess fatty acid-fed aging mice. The mononuclear cell population of young-SO fed mice (Ly6C^high^; 8.9±1.8%) were compared with aging-SO fed mice (15.8±1.3%) (Figure 5D). Thus, excess omega-6 enriched SO diet promotes inflammation in aging, marked by an increase in Ly6C^high^ population post-MI.

### Excess fatty acid intake in aging mice displayed dual population of CD11b^+^/F4/80^+^ cells in infarcted LV post-MI

LV analyses of CD45^+^/CD11b^+^ by flow cytometry showed no difference in the percentage population LC fed young and aging mice in no-MI controls. The SO fed aging mice displayed higher percentage of CD45^+^/CD11b^+^ (12.3%) cells compared to young-SO (1.9%) in no-MI controls (Supplementary Figure 8E). Post-MI, there was a global increase in CD45^+^/CD11b^+^ population due to excess intake of omega-6 fatty acids. The SO fed young and aging mice displaying high percentage of CD11b^+^ cells i.e. 29.5±4.1% and 38.9±3.4%, (Figure 5E and 5G) compared to LC-fed young and aging cohorts, respectively. We noted a signature population in the LV infarct of aging mice. The infarcted LV of SO fed aging mice showed the dual population of macrophages based on CD11b expression. At d0, naïve controls and young-LC mice, displayed a single population of CD11b^+^ cells, but aging LV displayed dual population CD11b^low^/F4/80^high^ and CD11b^high^F4/80^high^ cells (Supplementary Figure 8H). The young-SO mice showed a 3-fold increase in the expression of CD11b^+^F4/80^+^ compared with young-LC pre and post-MI. Post-MI, both LC and SO-fed aging mice did not show any change CD11b^high^/F4/80^high^ percentage population (Figure 5F). There was a major increase in CD11b^low^F4/80^high^ (11.82±2.6%) cells in aging-SO mice compared with aging-LC (6.32±3.4%) mice (Figure 5G and H). Thus, excess omega-6 diet aggravates macrophage population and expands them to a dual macrophage population in aging post-MI.

### Excess omega-6 intake aggravated MI-induced cardiorenal inflammation

**Figure 6:**
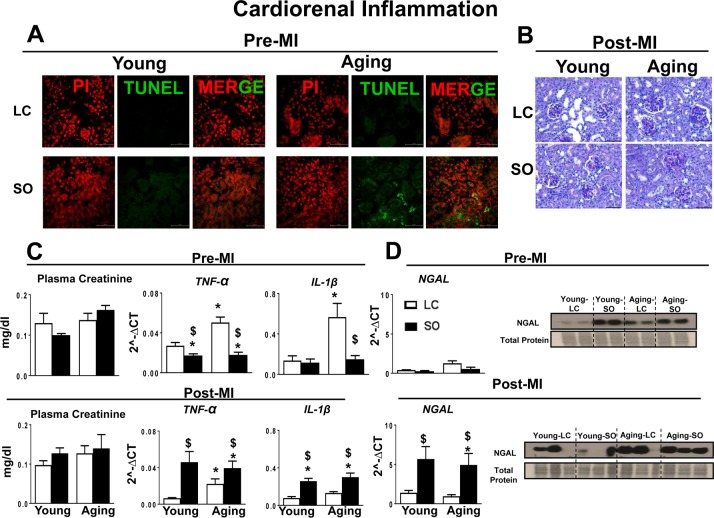
Post-MI impact of fatty acids during aging on cardiorenal-axis (**A**) Immunofluorescence images representing TUNEL positive cells (green) in young and aging-SO fed mice. Nuclei are stained with propidium iodide (Red). (**B**) PAS staining indicates granulomatous kidney inflammation. (**C**) Pre- and post-MI plasma creatinine level and mRNA expression of *TNF-α and IL-1β*, in kidney. (**D**) Pre- and post-MI mRNA and protein expression of *NGAL* in kidney. *p<0.05 vs young-LC; $p<0.05 LC vs SO. Values are means ±SEM; n=; n=2 at d0, n=3-4 at d1/group.

MI-induced cardiorenal inflammation is a multifactorial situation in which the LV and kidney are simultaneous-ly affected and their deleterious effects are reinforced in a feedback cycle that aggravates progression of HF [18]. Thus, we evaluated the markers of cardiorenal inflammation in aging for the post-MI setting with and without excess fatty acid intake. Pre-MI, apoptosis in the kidney was examined by TUNEL method. The standard diet fed young-LC and aging-LC mice did not display any TUNEL positive cells. The excess intake of omega-6 fatty acids increased TUNEL positive cells in aging kidney, but not in young kidney (Figure 6A). Compared to LC control, SO-fed aging mice showed glomerulus expansion with elevated inflammation (Figure 6B). Excess intake of fatty acid increased plasma creatinine levels in SO-fed aging mice indicated renal inflammation (Figure 6C). Post-MI renal inflammation due to excess intake of fatty acid in young and aging mice was confirmed by upregulation of renal injury and inflammation markers such as *NGAL* (4.5 fold young SO; 5.7 fold aging SO; p<0.01)*, Tnf-α* (6.6 fold young SO; 1.7 fold aging SO; p<0.01), and *IL-1β* (3.3 fold young SO; 2.2 fold aging SO; p<0.01) compared with respective young LC pre-MI and at d1 post-MI (Figure 6C and D).

### Aging decreased LV healing markers post-MI with excess intake of omega 6 fatty acids

Post-MI receptor (FPR2/ALX) based biomolecule(s) action and enzyme activation (5-LOX) with resolving gene markers (*Arg-1* and *Ym-1*) expression are essential for early LV healing. Post-MI early expression of these markers indicates resolution of inflammation. In no-MI controls (d0), the FPR2/ALX and 5-LOX protein expression was increased as an impact of aging (aging-LC and aging-SO mice). Post-MI, SO-fed aging group showed decreased FPR2/ALX expression (FPR2/ALX-0.5 fold, p<0.05 and 5-LOX -;1.5 fold, p<0.05) compared with young-SO group. The excess influx of fatty acid in aging decreased the expression of pro-resolving markers *Arg-1* (2.7 fold; p<0.05) and *Ym-1* (2.6 fold; p<0.05) in infarcted LV compared with young-SO group (Supplementary Figure 2A and B). Thus, excess intake of omega-6 fatty acids dysregulated the infarcted LV healing markers post-MI in aging.

## DISCUSSION

**Figure 7:**
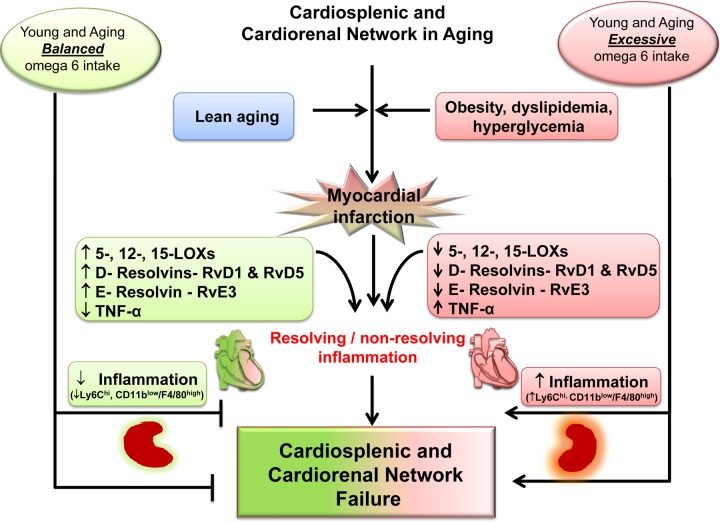
Schematic summary indicating the dysregulation of MI-induced cardiorenal and cardiosplenic network in aging.

Non-resolving, overactive inflammation is the main cause of post-MI ventricular dysfunction transition to HF [5, 8]. Thus, it is essential to understand how lipid signaling and molecular pathways are interlinked in the non-resolving inflammatory milieu to develop a novel therapeutic strategy for the treatment and prevention of HF [8]. Here, we provide generation of a lipid metabolite pathway that was previously technologically unfeasible, showing the ability of LOX enzymes to metabolize omega-6 fatty acids, which differentially modify the leukocyte phenotype in young and aging post-MI. With the application of LC-MS/MS technology for the analyses of lipid mediators and flow cytometry for analyses of immune cells, we revealed that excess intake of omega-6 fatty acids: 1) decreases LOXs activity to metabolize omega-6 fatty acids and promotes inflammation in aging; 2) increases AA-metabolites in only young; 3) reduces specialized proresolving D- and E-series resolvin biomolecules and their precursors in young and aging; 4) increases CD11b^low^F4/80^high^ population in infarcted LV in aging mice; and 5) drives cardiorenal inflammation in young and aging (Figure 7). Moreover, we show that the non-resolving trigger is substrate-dependent (quality and quantity) that determines chemokine/eicosanoids/docosanoids signaling and drives cytokine signaling in HF pathology, indicated by increased TNF-ɑ only in aging post-MI. Despite the essential nature of omega-6 fatty acids for health and homeostasis, in post-MI edematous milieu, the most likely scenario is that the lack of formation of biomolecules in aging drives the non-resolving inflammation that drives HF with increased leukocytes (e.g. ↑Ly6C^high^ and ↑CD11b^low^F4/80^high^) in the infarcted LV (Figure 7).

### Dysregulation of D- and E-series resolvins thereby amplified inflammation post-MI

In the clinical setting, post-MI injury leads to the development of LV dysfunction, as well as histological and structural changes that account for HF due to reperfusion therapy, but the triggers of this unknown chemical remodeling presented a challenge in the 20^th^ century [19]. Using lipidomics technology, we measured the AA, DHA, and EPA metabololipidome in young and aging mice in relevance to excess omega-6 intake in a post-MI setting. We showed LOX (-5,-12, and -15) mRNA expression was decreased in aging. Likewise, docosapentaenoic acid metabololipidome was also impacted in response to excess influx of omega-6 fatty acids. As the LOXs catalyze the transformation of free arachidonic acid into several pro-inflammatory and pro-resolving products, the decrease in LOXs during aging reduces the ability to form several eicosanoids/docosanoids, which are essential to resolve post-MI inflammation. Omega-6-derived products (HETEs, HODEs, prostaglandins; PGs, thromboxanes; TXs and leukotrienes; LTs and lipoxins) act as first line chemoattractants/defenders during the acute inflammatory response. LOXs also metabolize omega-3 fatty acids into pro-resolving products (HEPEs, monohydroxy-DHA and -EPA, further D- and E- series resolvins) that turn on resolving mechanisms overlapping with the acute inflammatory response [20]. Thus, an overall decrease LOXs expression in aging imbalances the overall synchronous and transitional cycle of the acute response to resolve inflammation post-MI. The post-MI acute response activates PGs and LTs, which are critical for wound healing, [8, 21] and also overlap with tissue repair to activate the resolution of inflammation process carried out by endogenously biosynthesized chemical mediators such as lipoxins, resolvins, protectins, and maresins [10, 21]. Excess omega-6 fatty acid intake decreased specialized pro-resolving molecules like D- and E-series resolvins (e.g RvD1, RvD5, RvE3) in young and aging mice. As a result of lowered D-and E-series resolvins and their precursors, there is a delay in the effective clearance of leukocytes with increased levels of the cytokine, TNF-ɑ. Thus, imbalance of post-MI chemokine signaling implicates the dysregulation of acute inflammatory response in LV healing post-MI with intake of omega-6 fatty acids.

The role of essential omega-6 and conditional omega-3 fatty acids is well-established and they are important biomarkers in HF and coronary heart disease [22-24]. The excess influx of omega-6 fatty acids in young mice robustly increased the levels of AA eicosanoids pre- and post-MI, but the aged mice failed to generate a similar kind of response towards AA eicosanoidome post-MI. In post-MI injury PGs, TXs and LTs are potent chemotactic factors for leukocytes (neutrophils, eosinophils and mononuclear cells) [21, 22, 25]. Cyclooxygenase (COX) -1 and -2 drives formation of these molecules, but also forms prostacyclin having antithrombotic property, therefore the inhibition of COXs can induce MI events [25, 26]. With adverse events from COX-2 inhibitors, it is understood that formation of PGs, TXs, and LTs is essential to initiate the post-MI acute inflammatory response in the infarcted area. AA metabolizes to PGs, TXs and LTs, which aggravate pro-inflammatory response post-MI and at same time, also forms epoxyeicosatrienoic acid (5-6-, 8-9, 11-12, 14-15 EETs) to balance inflammation with activation of the cytochrome P450 epoxygenase pathway [27]. Conventionally, PGs are considered pro-inflammatory, but recent evidence suggests that PGE_2_ acts as a stemoattractant, which promotes cardio-myocyte replacement post-MI. In a mesenchymal stem cell transplant study, PGE_2_ downregulated leukocyte proliferation *in-vitro*, decreased the number of cytotoxic CD8^+^T cells in the infarcted myocardium, preserved immune privilege, and restored cardiac function post-MI [28, 29]. Thus, these first-line defenders are essential during the acute phase of MI response, but the interaction between the overabundance of substrate and aging alters all lipid bioactive species and LOX efficiency in LV healing post-MI. This demands long-term studies to determine the mechanisms of HF in aging.

### Splenocardiac and cardiospelnic link in post-MI healing

Traditionally, ischemic heart disease is studied in a vascular and in a cardiocentric manner. Recent evidence suggest that MI-stimulates leukopoiesis, both in the bone marrow and at the extramedullary splenic site [8, 30, 31]. In response to MI, 40% to 75% splenic monocytes enter the blood stream and mobilize to the infarct region to govern ischemic myocardium healing. Clinical imaging confirms the translation of the splenocardiac axis and metabolic activity using ^18^F-fluorodeoxyglucose, suggesting the link of metabolism and inflammation in ischemic heart disease [30-32]. Our previous report with chronic use of omega-6 fatty acids promotes bone marrow adiposity in aging mice, supporting the outcome of presented findings [11, 33]. Of note, there is dissension in the literature for the source macrophages post-MI, which is either the yolk sac and/or fetal liver [34] versus monocyte-derived macrophages from the LV injury site replaces residential macrophages with age [35]. Practically, the use of fate mapping studies has limited translational value because of the limited availability of human hematopoietic, spleen and LV samples in a time dependent manner for post-MI studies. The age-induced dysregulation in the innate immune system has been well established [36, 37]. The innate immune response substantially alters with age, especially through dys-regulation of pro-inflammatory cytokines such as interleukin (IL)-6, IL-1β, TNF-α, and TGFβ, which lead to chronic inflammation, and thus contribute to the inflammaging phenotype, often observed in the elderly [38-40]. The leukocyte response in both young and aging groups showed high levels of Ly6G^+^ (neutrophils) and Ly6C^high^ (pro-inflammatory macrophages) population that resist to clear due to an altered chemo-kine microenvironment that expands the innate phase post-MI. Interestingly, the aging mice displayed a distinct macrophage population (F4/80^+^) with high levels of CD11b expression called (CD11b^high^). Since CD11b expression is commonly associated with neutrophils recruitment and monocytes, [26] the persistent presence of F4/80^+^/CD11b^high^ population in aging mice without MI indicated a pro-inflammatory environment results in low-grade inflammation and this phenomenon has been termed as “inflammaging” [41] is the primary cause of recurrent MI in HF. Young mice fed excess fatty acids displayed higher expression of CD11b compared with standard diet fed young mice post-MI. This possibly could be due to an increase in the number of AA eicosanoids within the milieu. Thus, lifestyle-related post-MI setting opens a new avenue and exciting opportunity to focus on post-MI eicosa-noids or docosanoids levels and their leukocytic receptors in future perspective studies to prevent the progression of HF.

### Post-MI cardiorenal syndrome in aging

Reports indicate that MI-induced advanced systemic inflammation and renal dysfunction are accompanied by worsening of renal failure leading to heart failure, and mortality [42, 43, 18]. Our study suggests that an excess fatty acid-enriched diet is the primary factor in renal inflammation pre-and post-MI, with an observed adaptive response in young and a maladaptive response in aging groups. The elevated levels of plasma creatinine in aging indicated the presence of renal inflammation. The post-MI cardiorenal syndrome is also present at the young age, however it cannot be detected by plasma creatinine, as it is an unreliable indicator of acute kidney injury. NGAL is commonly used as an immediate marker of acute kidney inflam-mation [44, 45]. The immediate increase in NGAL expression post-MI in the kidney in young age samples with elevated levels of pro-inflammatory markers TNF-α and IL-6 established the presence of cardiorenal syndrome. Excess omega-6 fed mice showed renal inflammation in aging is evident with an increase in NGAL expression prior to MI. Thus, supplementation of excess fatty acids aggravated post-MI to kidney injury through further increasing the level of the pro-inflammatory chemokines and marked increase in glomeruli apoptosis in aging.

### Clinical translational perspective

Maintaining the optimal balance of inflammation is crucial to successful post-MI LV healing [8]. Failure to maintain this balance contributes to HF, which is characterized by persistent inflammation and failed resolution influenced by lowered levels of pro-resolving molecules like D- and E-series resolvins and lipoxins [13]. In coronary heart disease and HF, the ratio of pro-resolving mediators (D-series resolvins, E-series resolvin, EETs) to pro-inflammatory mediators (PGs, LTB_4_, TXB_2,_ tetranor-12-HETE) becomes imbalanced and leads to non-resolving inflammation [8, 13, 27]. Although the mechanisms of the specific cell type and receptor-interaction of these molecules in HF remain to be elucidated, it is possible that some resolvins (e.g. RvD1) may play a role through the activation of leukocyte FPR2 receptor, as we described in our previous report [5]. Of note, omega-3 fatty acids are known for their cardiovascular benefit (1g/day) or to reduce elevated triglycerides using higher doses (3-4 g/day) [46]. Although, the reports cannot be ignored that indicate no benefit or rebuke cardiovascular benefit that may stem from many factors such as aging, excess omega-6 intake, anti-inflammatory treatment or combination of these, which serve to modulate LOXs lipid busting capacity, which needs to be investigated in the future [26, 47, 48].

### Study limitations

One limitation was the mice strain and sex, as we studied only male C57BL/6J mice. Potentially lipid metabolism and post-MI lipid remodeling is distinct in female mice or other strains of mice with aging. Another was that we studied 24h hr day one time point post-MI. A time dependent long-term study would have allowed us to correlate with chronic HF of clinical setting. Further studies will be necessary to investigate level of eicosanoids and docosanoids and their influence on leukocyte phenotypes in time dependent manner in order to promote healing and reduce cardiac remodeling.

Taken together, age-associated decrease in lipid processing enzymes (5-,12- and 15LOX) dysregulates the formation pro-inflammatory and pro-resolving molecules depending on the influx or availability of substrate that tempers acute phase leading to promotion or repression of inflammation post-MI.

## METHODS

### Animal compliance

All animal procedures were completed in compliance with the “Guide for the Care and Use of Laboratory Animals” (8th Edition, 2011), AVMA Guidelines for the Euthanasia of Animals: (2013 Edition) and were thereby approved by the Institutional Animal Care and Use Committees at the University of Alabama at Birmingham, USA.

### Mice and coronary ligation surgery

C57BL/6J mice 6 months (young) and 18 months (aging) old were sourced from National Institute of Aging colony (NIH, USA) and were maintained with free access to water and diet under a constant temperature of 19.8 – 22.2°C. Young adult and aging mice were randomized into two groups and were 1) LC (fed on standard lab chow, American Institute of Nutrition 93M diet, 4% w/w fatty acids) and 2) SO (fed on 10% w/w safflower-enriched diet) for 2 months (Table 2). After 2 months, the young and aging groups with and without SO diet were subjected to coronary artery ligation surgery to induce MI (Fig. 1A, study design). Non-surgery mice were maintained as d0 naïve controls. To induce MI, mice were subjected to the surgical ligation of the left anterior descending coronary artery, as previously described [49]. The mice were monitored after surgery until MI-d1 for necropsy.

### LV function measurements using echocardiography

Vevo 770 (Visual Sonics Inc. Canada) in vivo imaging system was used in order to perform echocardiographic analysis with probes up to 40 MHz with a resolution of 30 μm. Prior to echocardiography, the mice were anesthetized using 1.5-2.0% isoflurane in a 100% oxygen mix. Using a surface electrocardiogram, the heart rates and electrocardiogram were monitored. Increased heart rates (heart rate >400 beats per minute) were maintained during the acquisition to achieve physiological measurements and short (M-mode) and long axis (B-mode) images of hearts were obtained. The measurements were taken in the mid-papillary region in two dimensional parasternal long-axis and short-axis recordings. Studies were performed prior to necropsy for d0 naïve control mice as well as d1 post-MI young and aging mice. Three images were obtained for each variable from consecutive cardiac cycles. They were averaged by operator-blinded to diet and age [49].

### Necropsy

Young and aging mice with and without MI were anesthetized under 2% isofluorane in 100% oxygen mix. Blood was collected from the carotid artery 5 minutes post-heparin (4 IU/g; I.P.) injection and was centrifuged in order to collect plasma. Plasma aliquots were snap-frozen and stored at −80°C for plasma analysis. Lungs were collected, weighted, and process-sed, as previously described. 2 To arrest the left and right ventricles in diastole, they were perfused with cardioplegic solution. The left ventricle was then dissected into three sections: (1) the remote region (LV control: LVC), (2) the infarct region (LVI), and (3) the LV mid-cavity. The LVC and LVI were stored for later biochemical and LV mid-cavity histological and immunohistochemistry (IHC) analysis. The spleen and kidney were dissected via incision in the peritoneal wall, were weighed and processed. The spleen was divided into two halves. One half was fixed in 10% zinc formalin for IHC and the other was flash frozen and stored at −80°C, and stored for later biochemical and IHC analysis. The left kidney was longitudinally dissected. One half of the left kidney was fixed in 10% zinc formalin for IHC and the other was fixed in Tissue-Tek® (Sakura Finetek, Torrence, CA, USA) for immunocytochemistry. The right kidney was flash frozen and stored at −80°C for later biochemical and IHC analysis [49].

### Preparation of splenic protein for mass spectroscopy

15 mg of spleen tissue from young and aging; LC and SO mice at day 1 post-MI was homogenized in 1:9 ratio with 1X PBS (pH 7.4) and centrifuged at 10,000 rpm for 5 min at 4°C. The supernatant was collected and protein was measured using Bradford kit (Biorad Inc.).

### LC-MS-MS analysis of splenic metabololipodomics

HPLC was performed on a C18 column (Luna, C18(2); 2.1×150 mm, 3 μm; Phenomenex), mounted on the Prominence XR HPLC system (Shimadzu, Kyoto, Japan). The mobile phase consisted of a gradient between solution A, methanol-water-acetonitrile (10:85:5 v/v/v), and solution B, methanol-water-acetonitrile (90:5:5 v/v/v), both containing 0.1% ammonium acetate. The gradient program with respect to the composition of solution B was as follows: 0–1 min, 50%; 1–8 min, 50–80%; 8–15 min, 80–95%; and 15–17 min, 95%. The flow rate was 0.2 ml/min. The HPLC column was fully equilibrated to initial conditions before each sample was injected. The HPLC eluate was directly introduced to the electrospray ionization source (TurboV) of the QTrap5500 mass analyzer (AB Sciex, Framingham, MA, USA) in the negative ion mode with the following settings: curtain gas, 35 psi; GS1, 35 psi; GS2, 65 psi; temperature, 600°C; ion spray voltage, −1500 V; collision gas, low; declustering potential, −60 V; and entrance potential, −7 V. The eluate was monitored by the multiple reaction monitoring (MRM) method, to detect unique molecular ion–daughter ion combinations for each of the 60 transitions with 8 ms dwell time for each transition and 5 ms settling time between scans. The total cycle time was 1.625 s. Optimized collisional energies (18–35 eV) and collision cell exit potentials (7–10 V) were used for each MRM transition. The data were collected with Analyst 1.5.2 software (AB Sciex), and the MRM transition chromatograms were quantitated by MultiQuant software (AB Sciex). Concentration of each detected analyte in splenic extract was calculated by dividing the detected quantities (in nanograms) with their corresponding molecular masses and reported as nanomolar. Under standardized conditions, the detection limits of most eicosanoids are ∼2 pg on the column, and the limit of quantitation is 5 pg at a signal-noise ratio of 3. Since the sample volume used was 200 μl, this condition translates to an assay sensitivity of 0.03 nM for an average molecular mass of 330 of the detected eicosanoids.

### Post-MI metabololipidomics analysis using heat maps, and principal component analysis

For generation of heat maps, the splenic-lipid mediators (LM) concentration from young-LC, young-SO, aging-LC and aging-SO from day 1 post-MI was normalized by geometric mean for statistical significance. The normalized values were used for generation of Venn diagram, hierarchical cluster, and heat maps using cluster 3.0 and java tree view software. Correlations between young-LC, young-SO, aging-LC and aging-SO groups was observed using principal component analysis. The correlation-matrix was done using Pearson (n) test to avoid inflating the impact of variables with high variances on the PCA [5, 50].

### Application of Z-score to AA, DHA and EPA metabolome

In order to calculate z-scores the LMs were averaged for young-LC, young-SO, aging-LC and aging-SO. Standard z-score indicated how many standard deviations a measured value is above or below the mean. The higher the absolute value of the z-score, the more the values deviates from the mean and it indicates the significance of change in concentration. We report the significance score (p<0.05) as the absolute value of the z-score, so a significantly eicosanoids/docosanoids might be up- or down-regulated in a sample.

### LV, spleen and kidney RNA isolation and real-time PCR

Post-necropsy, frozen LV (remote and infarct), spleen and kidney samples were processed for RNA extraction. Briefly, tissue was homogenized with a sonic dismembrator (Fisher Scientific) at amplitude between 10 and 100 Hz in 500 μL TRIzol (Invitrogen). RNA was then extracted and isolated. cDNA synthesis was performed using 2 μg of total RNA using SuperScript® VILO cDNA synthesis kit (Invitrogen, CA, USA). Quantitative PCR (qPCR) for Alox-5 (Mm01182747_m1), Alox-12 (Mm00545833_m1), Alox-15 (Mm00507789_m1), tnf-α (Mm00443258_m1), FPR2/ALX (Mm00484464_s1), il-1β (Mm01336189_m1), arg-1 (Mm00475988_m1), ym-1 (Mm00657889_m1), ccl2 (Mm00441242_m1), ngal (Mm01324470_m1), grp40 (Mm00809442_s1) was done using TaqMan probes (Applied Biosystems, CA, USA) on master cycler ABI, 7900 HT. Gene levels were normalized to hypoxanthine phosphoribosyl-transferase (Hprt-1; Mm03024075_m1). Data was reported and analyzed as 2-ΔCT (ΔΔCT) values.

### Protein extraction and immunoblotting

LV infarct tissues were weighted and placed in their respective 1.5 mL tubes with 16 μl of 1xPBS (without calcium, Invitrogen) and 1x proteinase inhibitor (Roche Diagnostics). While at 4°C, the samples were dis-membrated in short, 10 second intervals using a sonic dismembrator until homogenous, then centrifuged at maximum speed (14,000 rpm). The supernatant was used as the soluble protein fraction. The kidney protein was extracted using RIPA buffer. Total protein determined with Bradford assay. Electrophoresis of 10 μg of protein from each tissue with XT bis-tris-4-12% gel (Bio-Rad Inc.) in MOPS buffer (Bio-Rad) was performed. Samples were then transferred onto nitrocellulose membrane (Bio-Rad Inc.) and a total protein stain was performed with Pierce reversible protein stain, nitrocellulose membrane kit (Thermo Scientific Inc.). Each membrane was blocked for 1 hour at room temperature with 5% non-fat milk powder (Bio-Rad) dissolved in PBS-T and probed with primary antibodies. Primary antibodies (FPR2/ALX 1:1000; Ccl2 1:5000; arg-1 1:5000, GPR40 1:1000; 5LOX 1:1000) were probed overnight at 4°C, subsequently washed with PBS-T, and a secondary antibody was applied (Biorad). Proteins were detected using Femto chemiluminescence detection system. Densitometry of protein blots were assessed with ImageJ software (NIH, USA).

### LV, spleen and blood flow cytometry

Single mononuclear cells were isolated from LV, blood and spleen from young-LC, young-SO, aging-LC and aging-SO mice post-MI d1 and were analyzed by flow cytometry with slight modification [4]. The cell count for LV mononuclear cells or splenocytes was adjusted to ∼1-2 million cells/stain. Isolated cell suspensions were finally suspended in 200 μl of 1:500Fc block and incubated for 10 min on ice. A cocktail of fluorophore-labeled monoclonal antibodies in 2X concentration were added for 30 min on ice as appropriate for each study. We used CD45-PE (BD Biosciences), CD11b-APC, F4/80-Percp (molecular probes), Ly6C-FITC (BD Biosciences), Ly6G-pacific blue (e-bioscience) in cocktail. All population are primarily gated using CD45+ markers for hematopoietic cells. Further, the neutrophils were defined as CD11b^+^/Ly6G^+^ cells. Activated macrophages were defined as the cells dual expression CD11b (Mac-1) and F4/80^+^surface marker. The monocytes/macrophages were also classified on the basis of CD11b expression as CD11b^low^/F4/80^high^ and CD11b^high^/F4/80^high^. The macrophages (F4/80^+^) were also classified as M1 (classically activated macro-phages) and M2 (alternatively activated macrophages) based on Ly6chigh and Ly6C^low^ respectively. Data were acquired on BDTM LSRII Flow Cytometer and analyzed with FlowJo software, version 7.6.3.

### Histological analysis of kidneys using PAS staining

Young and aging no-MI d0 naïve controls and post-MI, the kidneys were collected and weighed. Longitudinal middle slice of the kidney, taken through the hilum, was embedded in paraffin and stained with periodic acid-Schiff (PAS) reagent. In control and post-MI PAS-stained sections, glomeruli were systematically analyzed and levels of glomerulonephritis graded (from 0 to 4).4 Normal glomeruli were given a grade of 0, glomeruli with 1–25% glomerulonephritis were given a score of 1, those with 26–50% glomerulonephritis scored a 2, those with 51–75% glomerulonephritis scored a 3, and those with 76–100% glomerulonephritis scored 4.

### Statistical analyses

Data are expressed as mean and SEM. Statistical analyses were performed using GraphPad prism 5. Two-way analysis of variance (ANOVA) was used for comparisons between young-LC, young-SO, aging-LC and aging-SO. The basic parametric tests applied to metabolomics data and all the metabolites included in study were taken above the range of detection limit. Lipid metabolite below limit of detection excluded from the analysis to eliminate false discovery metabolite measurements. All immunoblotting densitometry data were normalized to total protein/lane. p<0.05 was considered as statistically significant.

## SUPPLEMENTARY MATERIAL FIGURES AND TABLE





## ABBREVIATIONS

AA: Arachidonic acidxs
CD: Cluster of differentiation
COX: Cyclooxygenase
DHA: Docosahexaenoic acid
DPA: Docosapentaenoic acid
EETs: Epoxyeicosatrie-noic acids
EPA: Eicosapentaenoic acid
HDHA: Hydroxydocosahexaenoic acid
HEPE: Hydroxyeico-sapentaenoic acid
HETEs: Hydroperoxyeicosatetrae-noic acid
HODEs: Hydroxyoctadecadienoic acids
HF: Heart Failure
IL-6: Interleukin 6
IL-1β: Interleukin-1beta
LC: Lab chow
LC-MS/MS: Liquid chromatography mass spectrometry
LOX: lipoxy-genase
LT: Leukotrienes
LV: Left ventricle
MI: Myocardial infarction
NGAL: Neutrophil gelatinase-associated lipocalin
PGs: Prostaglandins
Rv: Resolvin
SO: Safflower oil
TNF-α: tumor necrosis factor alpha
TX: Thromboxane
