# Supplementary material for: Aging dysregulates D- and E-series resolvins to modulate cardiosplenic and cardiorenal network following myocardial infarction
Source: Aging (Albany NY). 2016 Oct 18;8(11):2611–28. doi: 10.18632/aging.101077 (PMC5191859; doi:10.18632/aging.101077)
Supplement: Supplementary file 1 [file aging-08-2611-s001.pdf]

SUPPLEMENTARY MATERIAL

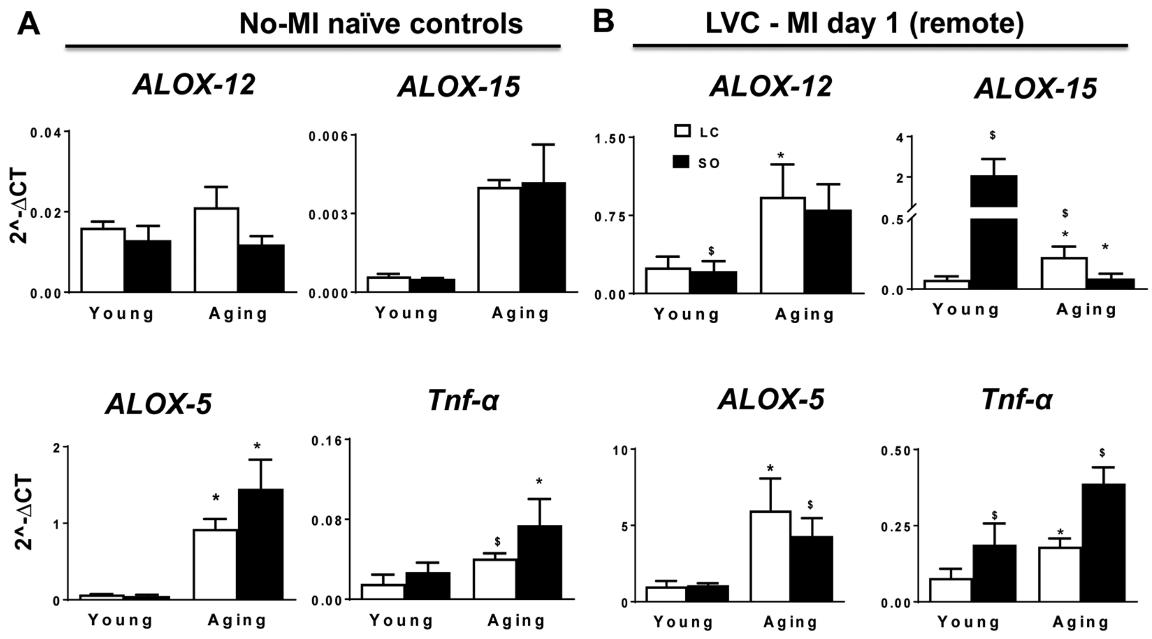

Figure S1. Excess omega-6 fatty acid influx impacted LOXs mRNA expression in young and aging mice. (**A**) mRNA expression of LOXs (5,12,15) and TNF-α in LV of no-MI naïve controls . (**B**) mRNA expression of LOXs (5,12,15) and TNF-α in remote myocardium (LVC) post-MI. *p<0.05 vs young-LC; $ p<0.05 LC vs SO. Values are means ±SEM; n=4/group.

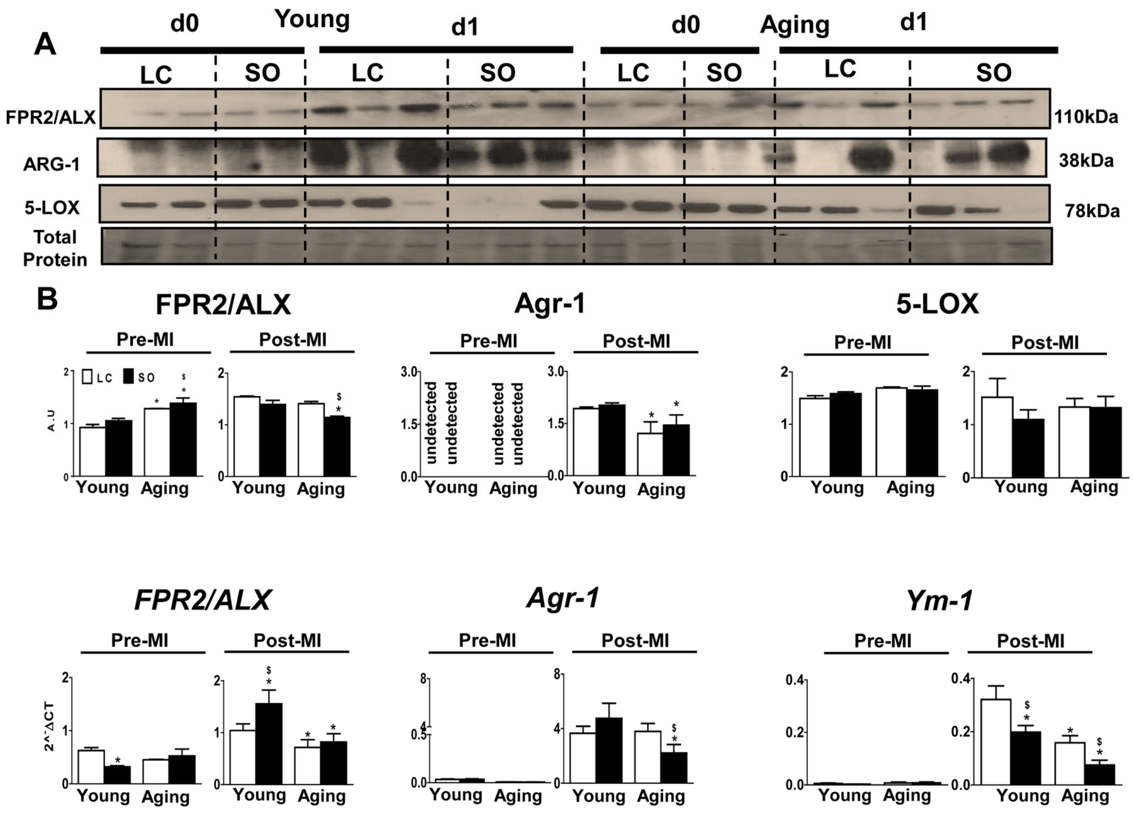

Figure S2. FPR2/ALX, 5-LOX and Arg-1 decreases post-MI due to excess intake of fatty acid during aging. (**A**) Immunoblot representing FPR-2/ALX, ARG-1 and 5-LOX protein expression at d0 control and post-MI d1 in young and aging mice with and without SO diet. (**B**) Pre- and post-MI densitometric analysis of mRNA expression for FPR2/ALX, ARG-1 and 5-LOX. Total protein is used as loading control; *p<0.05 vs young-LC; $ p<0.05 LC vs SO Values are means ±SEM; n=2 at d0, n=3-4at d1/group.

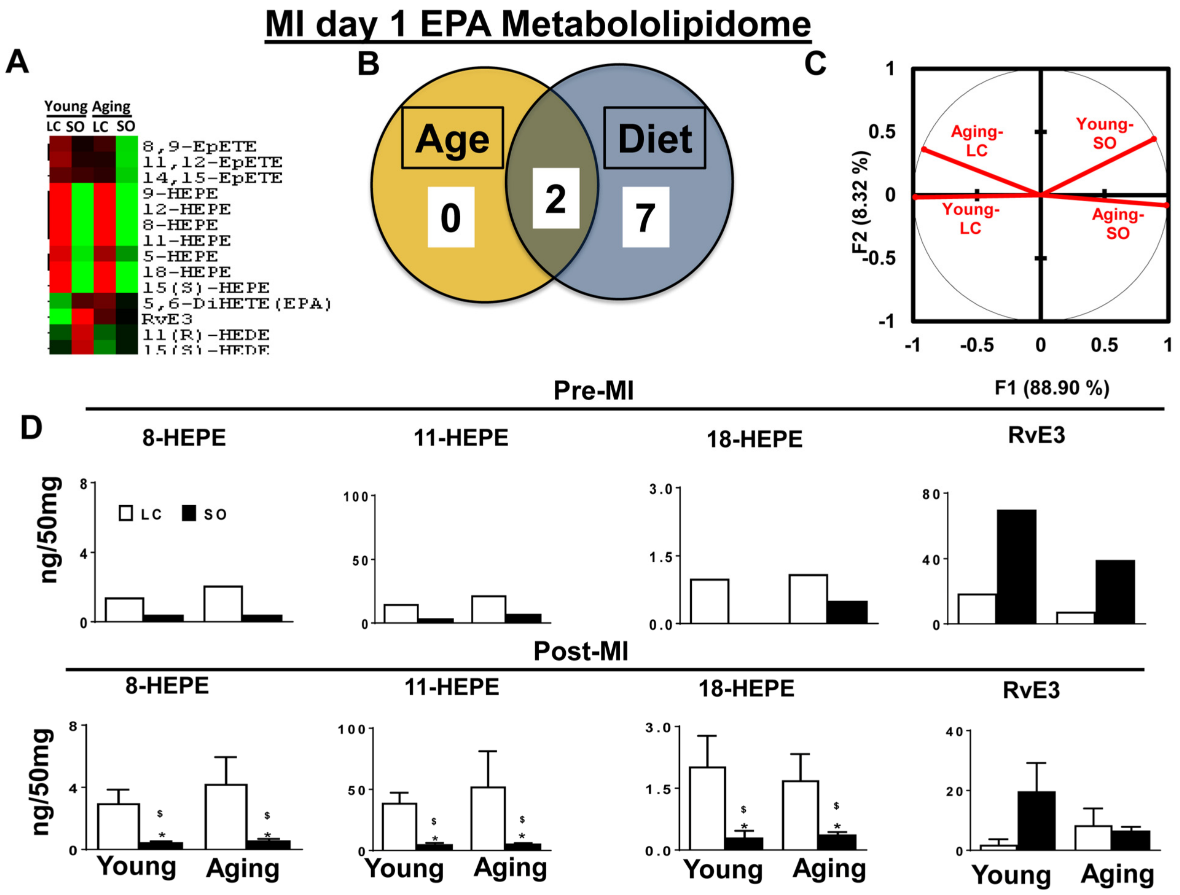

Figure S3. Post-MI dysregulation of EPA metabololipidome in response to age and excess omega-6 fatty acids intake. (**A**) Hierarchal cluster analysis of change in EPA metabolites due to young and aging, with and without SO diet. Color code bar representing change in expression from green (-1 lowest decrease) to red (+1 highest increase). (**B**) Venn diagram representing the number of EPA metabolites affected due to age (young and aging) and diet post-MI. (**C**) PCA analysis of EPA metabolites with respect to age and diet post-MI. (**D**) Bar graph representing change in EPA metabolite at pre-MI (No-MI) and d1 post-MI.

SUPPLEMENTARY TABLE

Please browse the Full Text version to see the data of **Supplementary Table 1.** Pre and post-MI lipid mediators levels in spleen (ng/ 50 mg spleen tissue).

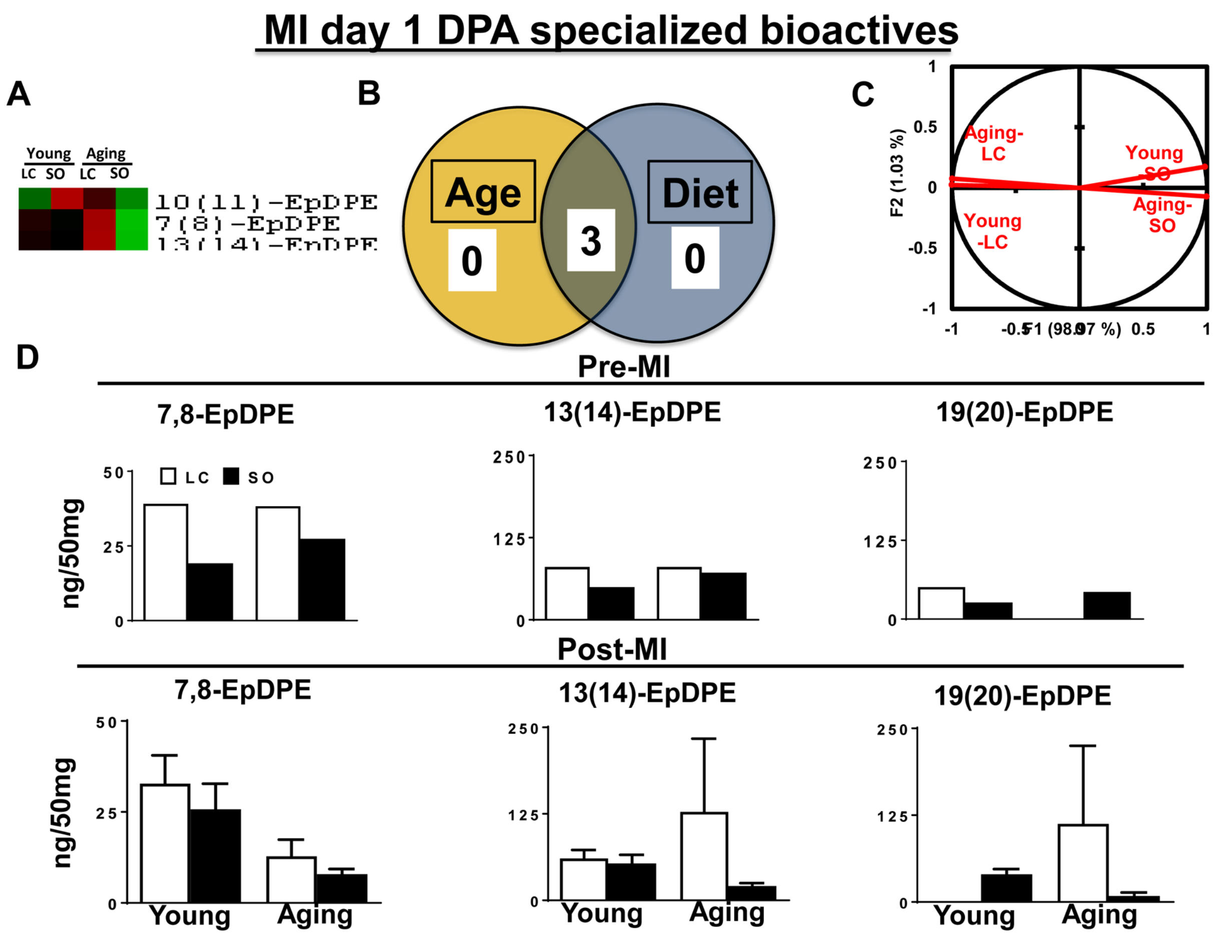

Figure S4. Post-MI changes in DPA metabololipidome profile in response to age and excess fatty acids influx. (**A**) Hierarchal cluster analysis of change in DPA metabolites due to young and aging, with and without SO diet. Color code bar representing change in expression from green (-1 lowest decrease) to red (+1 highest increase). (**B**) Venn diagram representing the number of DPA metabolites affected due to age (young and aging) and diet post-MI. (**C**) PCA analysis of DPA metabolites post-MI with respect to age and diet post-MI; n =3 mice per group. (**D**) Bar graph representing change in DPA metabolite at pre-MI (No-MI) and d1 post-MI.

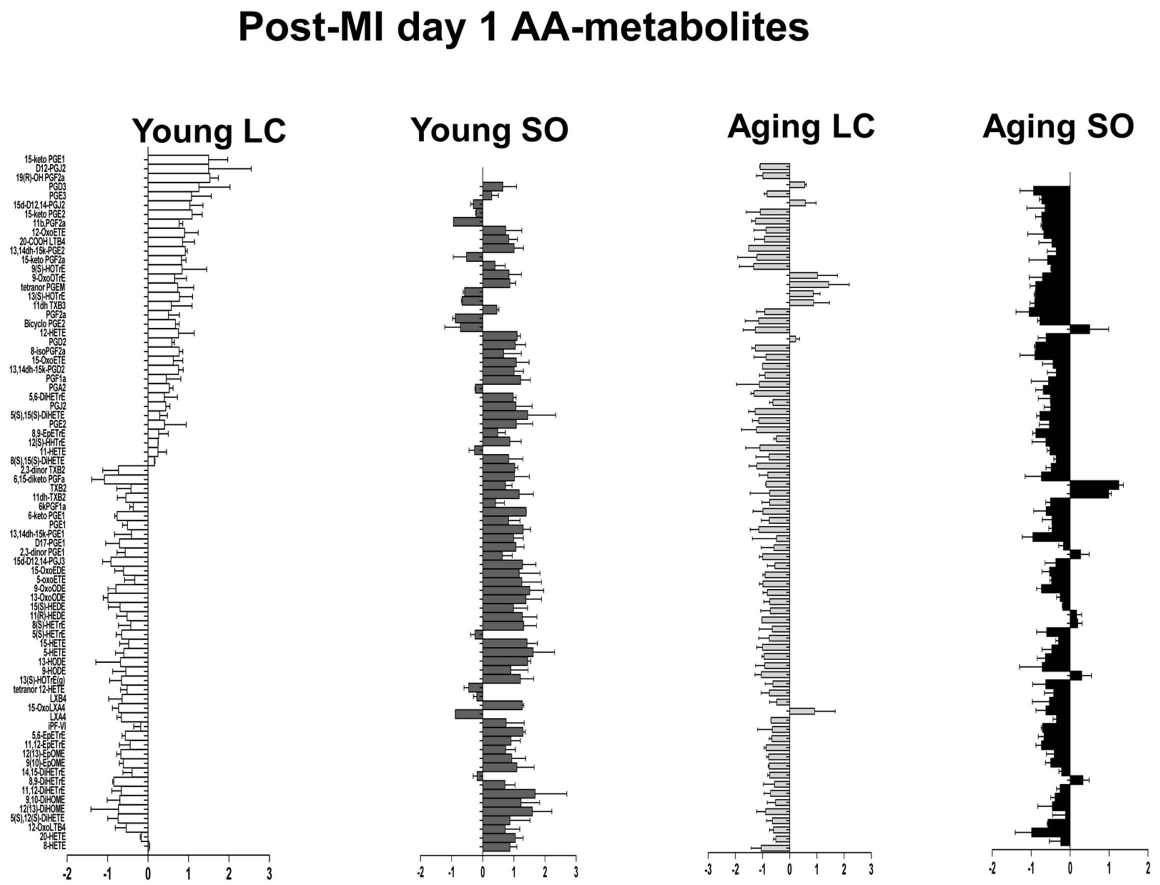

Figure S5. Z-score analysis of AA metabolites in young-LC, young-LC, aging-LC and aging-SO.

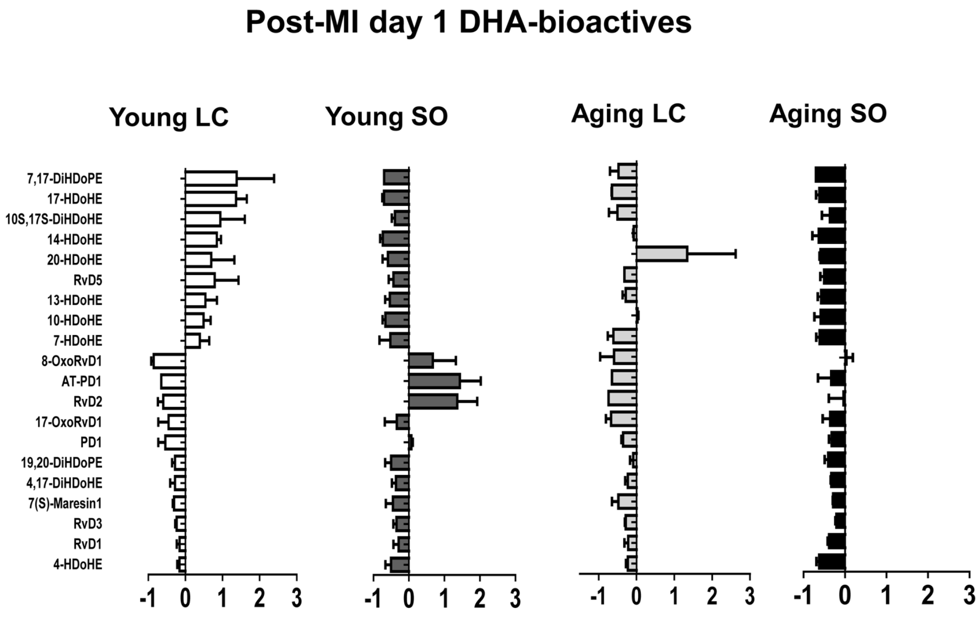

Figure S6. Z-score analysis of DHA metabolites in young-LC, young-LC, aging-LC and aging-SO.

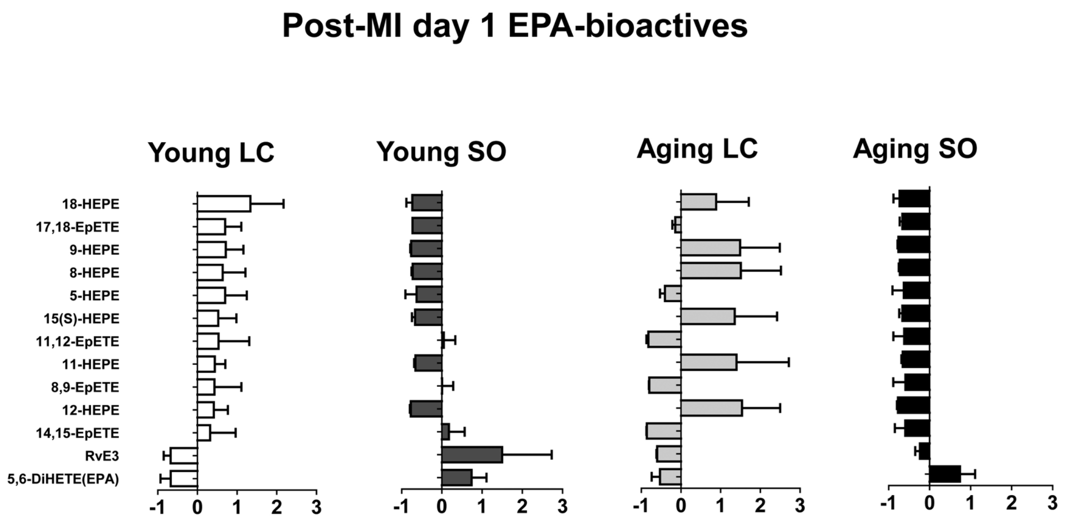

Figure S7. Z-score analysis of EPA metabolites in young-LC, young-LC, aging-LC and aging-SO.

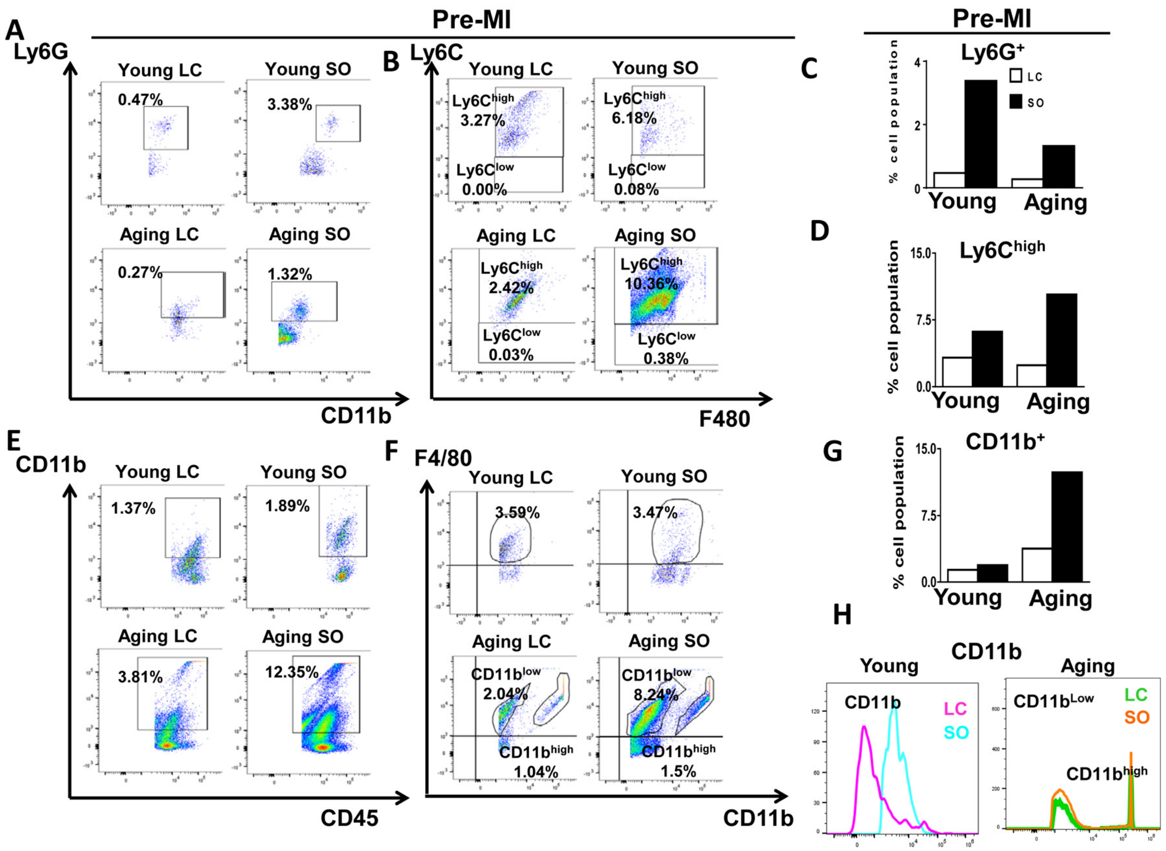

Figure S8. Excess intake of fatty acids increased F4/80+/Ly6Chigh and Ly6G+ population prior to MI. (**A**) Representative dot plots identifying the CD11b+ population in LV mononuclear cells isolated from LC and SO fed, young and old mice at d0 (NoMI). (**B**) Representative flow cytometry (FACs) dot plots showing Ly6Chigh in LV mononuclear cells isolated from LC and SO fed young and aging mice at d0 (No-MI). (**C**) Bar graphs representing percentage of Ly6G+ population in LV mononuclear cells at d0 (No-MI). (**D**) Bar graphs representing percentage of Ly6Chigh population in LV mononuclear cells at d0 (No MI). (**E**) Representative FACs dot plots showing CD45+/CD11b+ in LV mononuclear cells isolated from LC and SO fed young and mice at d0 (No-MI). (**F**) Representative FACs dot plots showing CD11blow/F4/80high and CD11bhigh/F4/80high in LV mononuclear cells isolated from LC and SO fed young mice at d0 (No-MI). (**G**) Bar graphs representing percentage of CD11b+ population in LV mononuclear cells at d0 (No-MI). (**H**) Histogram representing change in CD11b expression in young and aging mice at d0 (No-MI).

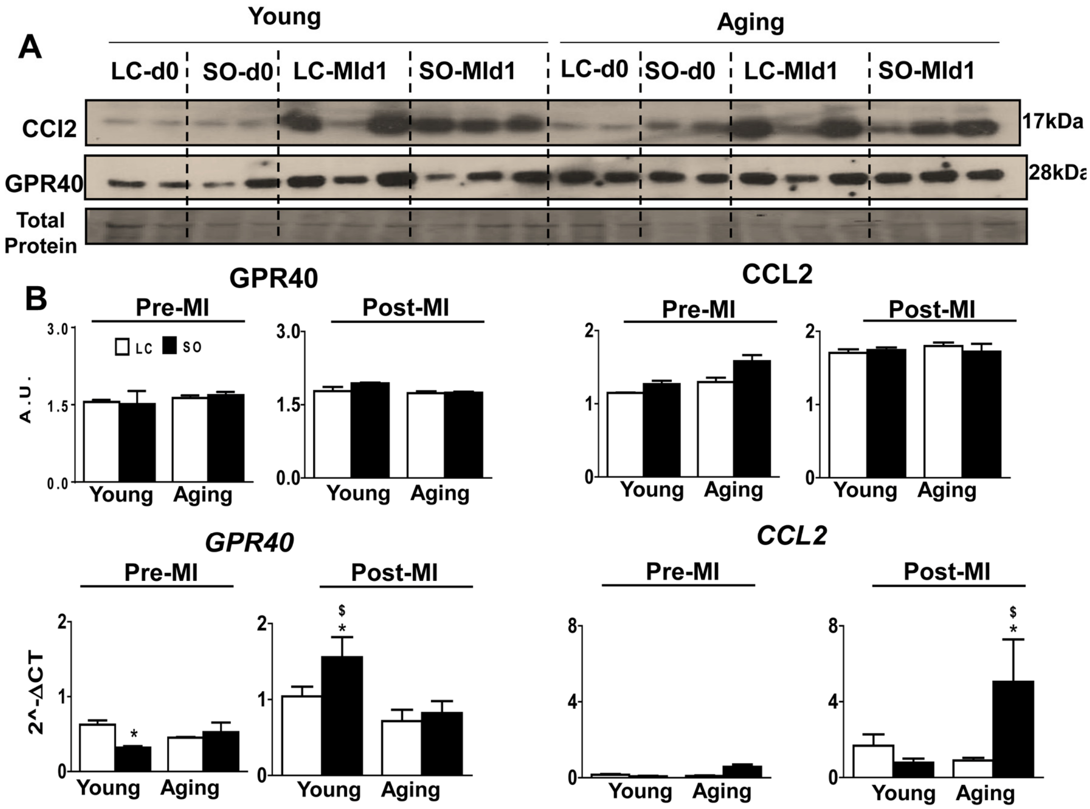

Figure S9. Altered expression of CCL2 and GPR40 post-MI in aging mice due to excess intake of omega-6 fatty acids in an infarcted LV. (**A**) Immunoblot representing CCL2 and GPR-40 protein expression at control (d0) and post-MI d1 in young and aging mice, with and without SO diet. (**B**) Pre- and post-MI densitometric analysis of CCL2 and GPR-40 immunoblots. Pre- and post-MI mRNA expression of CCL2 and GPR40 in LV. *p<0.05 vs Young-LC; $ p<0.05 LC vs SO. Values are means ±SEM; n=2-4/group.

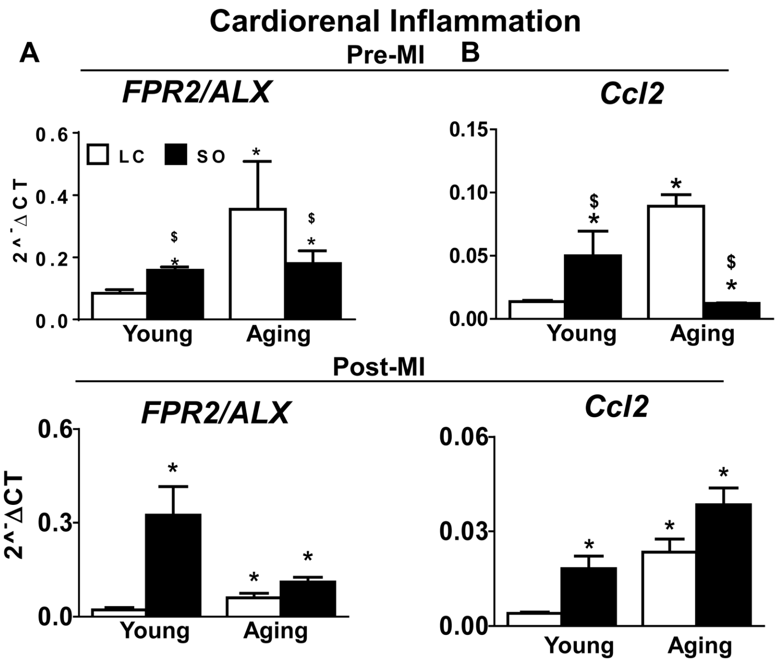

Figure S10. Effect of age and excess omega-6 fatty acids on pro-inflammatory gene expression in kidney post-MI d1. Pre- and post-MI mRNA expression of (**A**) FPR2/ALX in kidney (**B**) Ccl2 in kidney. *p<0.05 vs young-LC; $ p<0.05 LC vs SO. Values are means ±SEM; n=2-4/group.
